# Knockout of Multiple *Arabidopsis* Cation/H^+^ Exchangers Suggests Isoform-Specific Roles in Metal Stress Response, Germination and Seed Mineral Nutrition

**DOI:** 10.1371/journal.pone.0047455

**Published:** 2012-10-12

**Authors:** James M. Connorton, Rachel E. Webster, Ninghui Cheng, Jon K. Pittman

**Affiliations:** 1 Faculty of Life Sciences, The University of Manchester, Manchester, United Kingdom; 2 United States Department of Agriculture/Agricultural Research Service Children's Nutrition Research Center, Baylor College of Medicine, Houston, Texas, United States of America; United States Department of Agriculture, Agricultural Research Service, United States of America

## Abstract

Cation/H^+^ exchangers encoded by CAX genes play an important role in the vacuolar accumulation of metals including Ca^2+^ and Mn^2+^. *Arabidopsis thaliana CAX1* and *CAX3* have been previously shown to differ phylogenetically from *CAX2* but the physiological roles of these different transporters are still unclear. To examine the functions and the potential of redundancy between these three cation transporters, *cax1/cax2* and *cax2/cax3* double knockout mutants were generated and compared with wild type and *cax* single knockouts. These double mutants had equivalent metal stress responses to single *cax* mutants. Both *cax1* and *cax1/cax2* had increased tolerance to Mg stress, while *cax2* and *cax2/cax3* both had increased sensitivity to Mn stress. The *cax1/cax2* and *cax2/cax3* mutants did not exhibit the deleterious developmental phenotypes previously seen with the *cax1/cax3* mutant. However, these new double mutants did show alterations in seed germination, specifically a delay in germination time. These alterations correlated with changes in nutrient content within the seeds of the mutants, particularly the *cax1/cax2* mutant which had significantly higher seed content of Ca and Mn. This study indicates that the presence of these *Arabidopsis* CAX transporters is important for normal germination and infers a role for CAX proteins in metal homeostasis within the seed.

## Introduction

Metal transporters play a major role in regulating metal homeostasis, in controlling the acquisition of essential metal nutrients into the plant, coordinating the distribution and partitioning of these nutrients to appropriate locations within the plant and within individual cells, and preventing or responding to metal toxicity [1]. Studies of metal homeostasis over a number of years in model species like *Arabidopsis thaliana*, has led to the genetic identification of many metal transporters, a few of which have been characterised and understood in specific detail, but many of which are still poorly understood in terms of biochemical and/or physiological function [1]–[3]. In addition, far less is understood about the potential interactions and cross talk between metal transporters, and the influence that changes in one metal through the action of a specific transporter, have on the ionome, which defines the elemental composition of the whole organism [4].

CAX (cation/H^+^ exchanger) genes encode a family of important vacuolar-localised metal transporters that function as cation/H^+^ exchangers [5]. They mediate the high capacity sequestration of cations into the vacuole and are energised by the counter flux of H^+^ and thus utilise the large proton electrochemical gradient which exists across the tonoplast. The central vacuole of plant cells appears to be an important organelle for the regulation of metal homeostasis, both as a critical sub-cellular sink for providing metal tolerance and as an important source for bioavailable metals, such as during nutrient deficiency or for the propagation of Ca^2+^ signals [6]–[8]. Ca^2+^ transport is thought to be a major role of the plant CAX transporters, and all plant CAX genes examined to date have the ability to transport Ca^2+^ [9], [10]. Through the analysis of CAX mutants, vacuolar Ca^2+^/H^+^ exchange activity has been demonstrated to be a critical component in the maintenance of Ca nutrition. For example, knockout of *Arabidopsis* vacuolar Ca^2+^/H^+^ exchangers (*CAX1* and *CAX3*) can cause sensitivity to elevated Ca and other developmental phenotypes including reduced growth and inhibited stomatal function due to an inability of mesophyll cells to maintain low apoplastic Ca [11], [12]. In addition to a nutritional role, Ca^2+^ is critical for cellular signalling and has been shown to be required to mediate signalling events in response to abiotic and biotic stresses, and during development [13], [14]. Impaired hormone and developmental responses or abiotic stress phenotypes following deletion of *Arabidopsis* CAX genes such as *CAX1* and *CAX3*, therefore indicate Ca^2+^ signalling roles for these vacuolar Ca^2+^ transporters [11], [15]–[17].

Two of the six *Arabidopsis* CAX genes, *CAX1* and *CAX3*, are particularly important for Ca^2+^ transport and homeostasis. CAX1 alone accounts for a significant proportion of the total vacuolar Ca^2+^/H^+^ transport activity [15] but when *CAX1* is knocked out along with the most closely related gene *CAX3* (*cax1/cax3* mutant), the resulting plant is extremely sensitive to elevated Ca stress and has a very severe stunted phenotype [11]. However, single knockout mutants associated with these genes do not display such dramatic phenotypes, and it is not fully clear whether the phenotypes associated with the *cax1* and *cax3* mutants are solely due to impaired Ca homeostasis. Analysis of CAX proteins by heterologous expression has demonstrated that in addition to Ca^2+^, different CAX isoforms can transport other transition metals [18]–[24]. For example, *Arabidopsis* CAX2 can transport Cd^2+^, Mn^2+^, and Zn^2+^ when expressed either in yeast or tobacco [19], [21], while a *cax2* knockout has a significant reduction in vacuolar Mn^2+^ sequestration compared to wild type, but has no significant change in Ca^2+^ sequestration [25]. CAX1 and CAX3 may also be able to transport the monovalent cations Na^+^ and Li^+^ [26] while a *cax3* knockout has increased sensitivity to elevated concentrations of these ions [17]. In addition, expression of the CAX1 open reading frame in yeast found that it also has the ability to transport Mn^2+^ [27]. Other changes in metal sensitivity and content in CAX mutant plants appear to be due to indirect effects. For example, deletion of *CAX1* has been linked with an increased tolerance to Mg stress [15], [28] that is not due to a direct Mg^2+^ transport by CAX1 but possibly due to the relationship between Ca and Mg in plants [29]. Deletion of *CAX1* and *CAX3* also gives rise to changes in inorganic phosphate (Pi) mobilisation within the plant which is thought to be due to alterations in CAX-mediated signalling controlling Pi homeostasis [30].

Phylogenetic analyses have demonstrated that higher plant CAX genes are divided into two sub-groups, named Type 1-A and Type 1-B [9], [31]. *Arabidopsis CAX1*, *CAX3* and *CAX4* are grouped within Type 1-A, while *CAX2*, *CAX5* and *CAX6* are within Type 1-B. The relevance of these distinct groupings is unclear and so far, no clear-cut functional differences between the Type 1-A and Type 1-B CAX genes have been determined. The generation of the *cax1/cax3* double knockout mutant has allowed the examination of the genetic interactions, isoform specificity and redundancy of CAX transporters within the Type 1-A sub-group [11], [12], [26], but the potential interactions and possibility of redundancy by CAX genes between the Type 1-A and Type 1-B sub-groups have yet to be explored. To address this, *cax1/cax2* and *cax2/cax3* double knockout mutants have been generated in this study and were phenotypically compared alongside the *cax1*, *cax2* and *cax3* single mutants and wild type *Arabidopsis* plants under non-stressed and metal stress conditions. *CAX1*, *CAX2* and *CAX3* genes are known to be expressed in *Arabidopsis* seed [32] and it has recently been indicated that CAX transporters are involved in determining metal partitioning within the seed [33], but the physiological consequence of altered seed metal content following CAX mutation has not previously been studied. Seed germination was therefore quantified in the single and double CAX mutant plants. We describe that *cax1/cax2* and *cax2/cax3* double mutants have alterations in seed germination compared to wild type and single mutants, specifically a delay in germination time, which correlate with changes in metal content within the seeds of the mutants.

## Materials and Methods

### Plant materials and generation of *cax1/cax2* and *cax2/cax3* knockout lines

**Figure 1:**
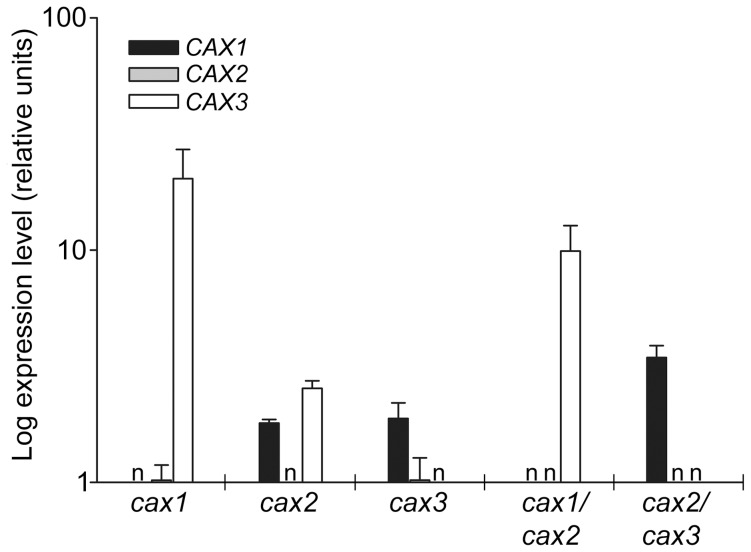
*CAX1*, *CAX2* and *CAX3* expression. Changes in *CAX* expression in *cax1*, *cax2* and *cax3* single and double knockout lines relative to Col-0 (wild type) were determined by real-time PCR using actin and ubiquitin as constitutive control primers. RNA was extracted from whole seedling tissue grown on 0.5×MS plates without additional metal supplementation for 21 d. Expression relative to actin is shown and expression relative to ubiquitin was equivalent. Relative fold changes in gene expression were calculated using the 2^−ΔΔCt^ method. Bars indicate the mean log expression±SE from three samples each replicated twice. ‘n’ denotes no increase in expression detected.

The *Arabidopsis thaliana* ecotype Columbia-0 (Col-0) was used for all experiments. Homozygous T-DNA insertion knockout lines *cax1-1* [15], *cax2-2* [25], *cax3-1* and *cax1/cax3* [11] were all in the Col-0 background. The *cax1/cax2* and *cax2/cax3* double knockout lines were generated by the crossing of the *cax1-1* and *cax2-2* alleles, and by the crossing of the *cax2-2* and *cax3-1* alleles, respectively, and the F_2_ generations were selected for double mutants. Homozygous double mutant alleles were identified by PCR using oligonucleotide primers generated previously for the selection of single homozygous mutants [11], [15], [25] and the knockout mutations were confirmed by real-time PCR gene expression analysis (Figure 1).

### Plant growth conditions

Wild type and mutant *Arabidopsis* were sown onto damp compost (William Sinclair Horticultural) in 9 cm diameter pots and placed in an environmental growth chamber (Percival Scientific) at 22°C with constant light, 160 µE m^−2^ s^−1^ light intensity, and controlled 70% relative humidity. For growth on solid media, seeds were surface sterilized with 90% (v/v) ethanol/30% (v/v) sodium hypochlorite then washed five times in Milli-Q water (Millipore) before plating on half-strength Murashige and Skoog (0.5×MS) basal medium (Duchefa) without sucrose, adjusted to pH 5.6 and containing 1% (w/v) agar, with or without added metal salts (25 mM CaCl_2_, 10 µM CdCl_2_, 10 mM LiCl, 50 mM NaCl, 25 mM MgCl_2_, or 1.5 mM MnCl_2_). Seeds were stratified for 2 d in the dark at 4°C before growth in an environmental growth chamber (Sanyo) at 22°C with a 16 h light/8 h dark cycle, 160 µE m^−2^ s^−1^ light intensity. For metal stress and fresh weight analysis, seedlings were grown following germination for 21 d. Entire seedlings were removed, blotted on tissue paper to remove excess water and weighed on a calibrated fine balance (Sartorius).

### Chlorophyll extraction

*Arabidopsis* seedlings were grown for 18 d on 0.5×MS plates supplemented with metals as described above. Shoot tissue was dissected from the seedling and fresh weight was determined. This was then ground on ice in 80% (v/v) acetone and centrifuged at 13 000×*g* for 5 min. The supernatant was recovered and the absorbance was measured at 647 nm, 664 nm and 750 nm. Total chlorophyll (chl a+b) content was determined using the equation: chl a+b (µg ml^−1^) = (17.76×*A*_647 nm−750 nm_)+(7.34×*A*_664 nm−750 nm_), as described by Porra *et al*. [34] and converted on the basis of fresh weight of tissue.

### Germination analysis

Seeds were combined from at least six homozygous parent plants and wild type plants, harvested at the same time and stored at room temperature for 3 months before sowing. Seeds from different lines of *Arabidopsis* were harvested at the same time and then sterilely sown onto 1% (w/v) agar (plant cell culture grade, Sigma-Aldrich, A7921) plates in a grid formation (approximately 60 per plate) and sealed with micropore tape. Seeds were stratified for 2 d in the dark at 4°C and then transferred to an incubator at 22°C with a 16 h light/8 h dark cycle. Seeds were left for 9 h and then observed every 2 h under a dissection microscope. Germination was scored using radicle protrusion from the seed coat as an indicator. The mean time to germinate or establish was determined by the equation: Σ(N*_t_*T) = /Σn, as described by Bewley and Black [35], where N*_t_* is the number of seeds germinated or established at each time point, T is the time point, and n is the total number of seeds. Seeds were left for 10 d and any that had not germinated in this period were determined to be non-viable.

### Real-time PCR analysis

Total RNA was extracted from *Arabidopsis* whole seedling tissue (three biological replicates of each line) grown on 0.5×MS plates without additional metal supplementation for 21 d as described above, using a Qiagen plant RNA extraction kit (Qiagen). DNase treated-RNA was converted to cDNA using Superscript III (Invitrogen) reverse transcriptase and an oligo(dT) primer. *CAX1*, *CAX2* and *CAX3* gene expression was determined by quantitative real-time PCR using a SYBR Green core qPCR kit (Eurogentec) and an ABI Prism 7000 machine (Applied Biosystems) using the SYBR Green detection program and normalised to actin *ACT2* and ubiquitin *UBC1* gene expression using primers QACT8L 5′-TGCAGACCGTATGAGCAAAG-3′, QACT8R 5′-CTGGAAAGTGCTGAGGGAAG-3′, QUBC1L 5′-TACCTCCATCCAGTCCTTGC-3′ and QUBC1R 5′-GCTCAACAACATCACGCACT-3′. Primers used for CAX gene expression were QCAX1L 5′-TCGCTGCTAGGTTCGATTTT-3′, QCAX1R 5′-GGCAGCAAGTGACACAAGAA-3′, QCAX2L 5′-TTGCCATGAAAGACAAGCTG-3′, QCAX2R 5′-CCGTCTCAAAAAGCTGGAAG-3′, QCAX3L 5′-GCGTTGGCCAATAACAAAGT-3′ and QCAX3R 5′-GGCGATACCACCAAAGAAGA-3′. Reactions were run in triplicate and PCR efficiencies checked using LinRegPCR [36]. Melting curves were produced for each experiment to ensure that single products were amplified. Fold change in gene expression in mutant lines was determined using the 2^−ΔΔCT^ method where ΔΔC_T_ = (C_T target gene_−C_T reference gene_)transgenic line−(C_T target gene_−C_T reference gene_)Col-0, as described [37].

### Metal content analysis

*Arabidopsis* leaves (from 4-week old plants) and seeds from plants grown on soil as described above without additional metal supplementation, were harvested for determination of metal content and oven dried at 60°C for 24 h. Approximately 15 mg dry weight per sample of leaf and seed material was then digested in 0.5 ml of ultra-pure concentrated nitric acid (67%) at 100°C for 3 h. Samples were diluted in Milli-Q water (Millipore) and analysed by inductively coupled plasma atomic emission spectroscopy (ICP-AES) (Perkin-Elmer Optima 5300) and calibrated using an internal standard solution, which was a matrix matched serial dilution of Specpure multi element plasma standard solution 4 (Alfa Aesae).

### Vacuolar-enriched membrane vesicle preparation, and proton and metal transport measurements

Vacuolar-enriched membrane vesicles were isolated from 2 week old *Arabidopsis* seedlings grown on 0.5×MS media, as described above. The seedlings were pre-treated with 50 mM CaCl_2_ and 1.5 mM MnCl_2_ 14 h before harvest. The procedure for plant tissue homogenisation in a mortar and pestle and preparation of microsomal membrane vesicles was as described previously [15], then vacuolar-enriched membrane vesicles were prepared following purification of microsomal vesicles through a two-step 22% (w/w) and 34% (w/w) sucrose gradient as described previously [38]. Membrane vesicle purity was determined by measuring ATPase and pyrophosphatase (PPase) hydrolytic activity in the presence of inhibitors of the tonoplast V-type H^+^-ATPase (V-ATPase), tonoplast K^+^-dependent H^+^-PPase, plasma membrane H^+^-ATPase and mitochondrial H^+^-ATPase, essentially as described previously [25], [39]. Tonoplast and mitochondrial ATPase activity was determined in the presence of 40 mM Tris-MES (pH 8.0), 50 mM KCl, 2 mM MgSO_4_, and 2 mM ATP, while plasma membrane ATPase activity was determined under the same conditions at pH 6.5. Tonoplast PPase activity was determined in the presence of 40 mM Tris-MES (pH 8.0), 50 mM KCl, 5 mM MgSO_4_, and 0.1 mM sodium pyrophosphate. The following inhibitors were used: 100 µM sodium orthovanadate (plasma membrane H^+^-ATPase inhibitor), 50 mM potassium nitrate and 0.2 µM bafilomycin (both tonoplast V-ATPase inhibitors), and 1 mM sodium azide (mitochondrial ATPase inhibitor). The absence of KCl was used as an inhibitor of tonoplast PPase activity. Fifty µl of tonoplast-enriched membrane vesicles (100 µg protein ml^−1^, quantified using a BioRad Bradford protein assay) were added to start the reaction and incubated at 37°C for 1 h, then the reaction was stopped by the addition of 1% (w/v) SDS. Inorganic phosphate (Pi) release as a measure of enzyme hydrolytic activity was determined by colorimetric assay following addition of reductant and colour developer solutions as described by Ohnishi *et al.* [40], then quantified by absorbance measurement at 720 nm.

Proton transport measurements in the purified membrane vesicles were performed to assess the V-ATPase activity in each genotype. V-ATPase H^+^ transport was measured by the fluorescence quenching of acridine orange to determine the formation of inside-acid pH gradient across the tonoplast vesicle membrane. Ca^2+^/H^+^ and Mn^2+^/H^+^ exchange activity was determined in the membrane vesicles by measuring the Ca^2+^- and Mn^2+^-dependent dissipation of the V-ATPase-dependent pH gradient by acridine orange fluorescence quench. The fluorescence quench assay was performed essentially as described previously [21], [41]. Membrane vesicles (50 µg protein) were added to a reaction buffer in a stirred cuvette containing 175 mM mannitol, 40 mM Tris-MES (pH 8.0), 50 mM KCl, 1 mM sodium azide, 0.1 mM sodium orthovanadate, and 5 µM acridine orange (Sigma), incubated at 25°C. The pH gradient was generated by energisation of the V-ATPase with the addition of 2 mM ATP and 2 mM MgSO_4_. The formation of the pH gradient was observed by monitoring the fluorescence quenching of acridine orange at excitation 495 nm and emission 540 nm using a Jasco FP750 fluorescence spectrometer. After a steady-state pH gradient was generated, 0.2 µM bafilomycin was added to inhibit V-ATPase activity then 200 µM of CaCl_2_ or MnCl_2_ were added and initial rate of fluorescence recovery measured. Analysis was conducted in duplicate or triplicate on each of three independently prepared membrane vesicles.

### Statistical analysis

Unless otherwise stated, all data shown is representative of at least three repeat experiments. Differences between treatments were assessed using one-way ANOVA or two-way ANOVA. When significant differences were detected at a 95% level of confidence, the multi-range Tukey's post-hoc test was applied. All statistical tests were performed using SPSS version 16 for Windows.

## Results

### Cation/H^+^ exchanger double knockouts display selective ion sensitivity phenotypes

A double mutant between the two closely related CAX genes *CAX1* and *CAX3* was previously shown to display significant growth impairment when grown on soil [11] or on basal nutrient solution [12]. To examine the potential phenotypes of double mutants generated between two phylogenetically distinct CAX genes; *CAX2* with either *CAX1* or *CAX3*, the homozygous *cax1-1*, *cax2-2* and *cax3-1* knockout mutants which we previously isolated and characterised [11], [15], [25] were crossed and *cax1/cax2* and *cax2/cax3* double mutants were selected in the F_2_ generation. Homozygous double mutant alleles were confirmed by PCR (data not shown), and lack of *CAX1* and *CAX2* expression (in *cax1/cax2*), and *CAX2* and *CAX3* expression (in *cax2/cax3*) was confirmed by real-time PCR (Figure 1). We previously found that knockout of *CAX1* leads to significant induction of *CAX3* expression [15]. The *cax2* and *cax3* mutants showed a moderate induction of other CAX genes, with *CAX1* and *CAX3* (in *cax2*) and *CAX1* (in *cax3*) induced slightly relative to wild type, while *cax1/cax2* and *cax2/cax3* displayed substantial induction of *CAX3* and *CAX1* expression, respectively (Figure 1). Induction of *CAX2* was not significantly higher than wild type levels in *cax1* and *cax3* lines.

**Figure 2:**
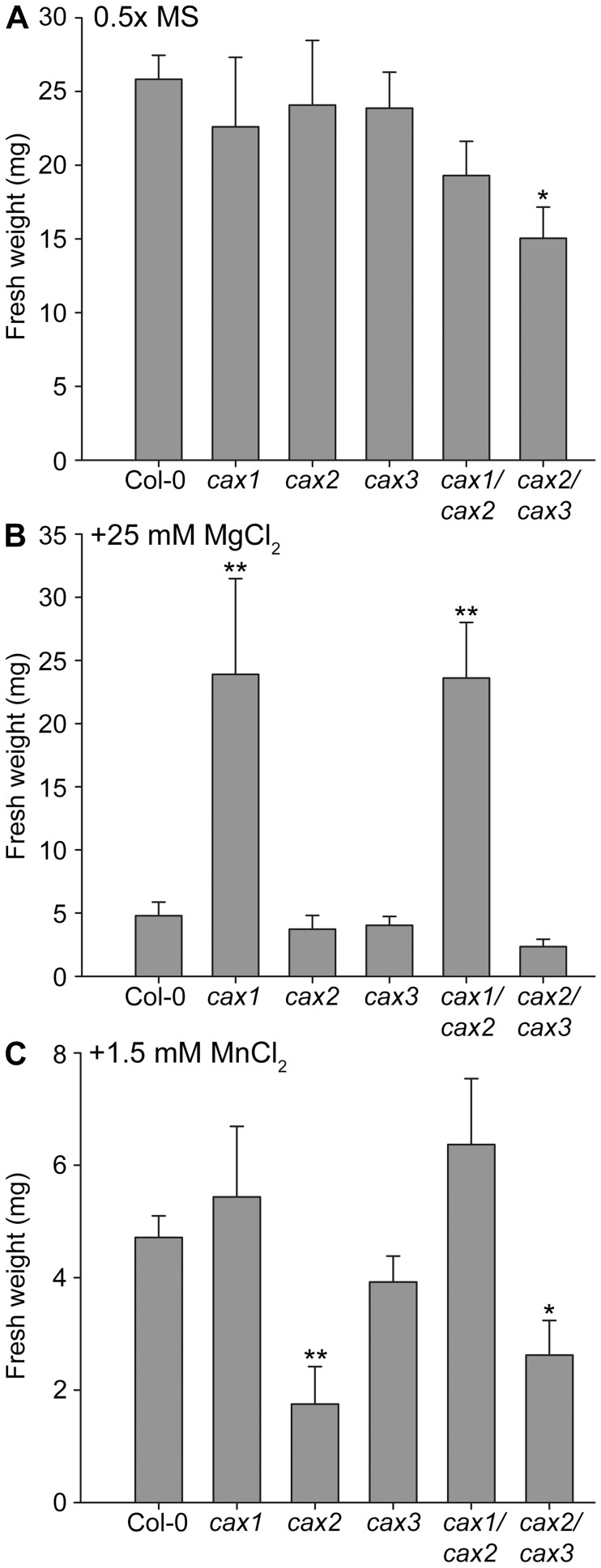
Mg and Mn sensitivity of CAX mutant plants. Fresh weight of Col-0 (wild type) and *cax* knockout mutant plants following germination and growth on solid 0.5×MS media (adjusted to pH 5.6) in the absence (**A**) or presence of 25 mM MgCl_2_ (**B**) or 1.5 mM MnCl_2_ (**C**). Bars indicate the mean±SE (*n* = 12–15) fresh weight measured in 21 d-old plants. ** (*P*<0.01) and * (*P*<0.05) denotes significant difference between CAX mutant lines and Col-0 control as determined by one-way ANOVA.

**Figure 3:**
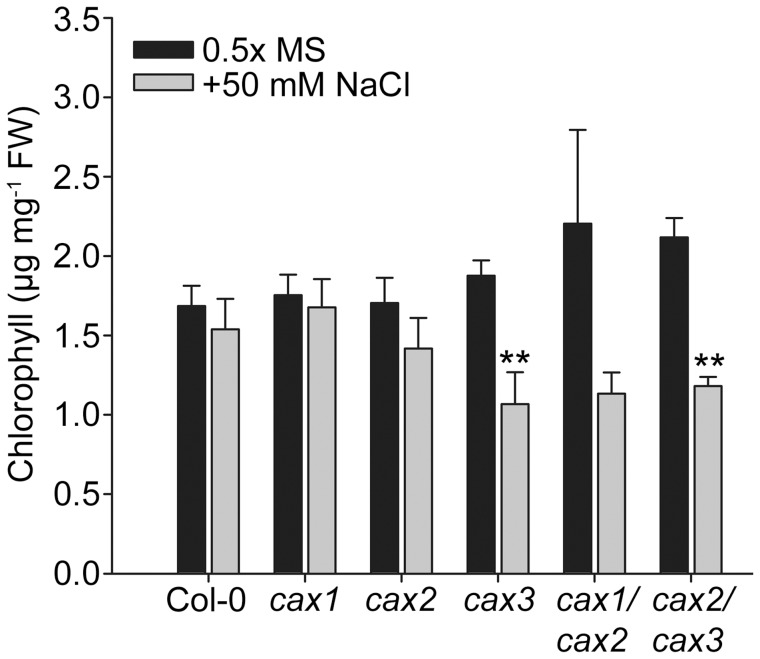
Na sensitivity of CAX mutant plants. Total chlorophyll content was measured in leaf and shoot tissue of 21 d-old Col-0 (wild type) and *cax* knockout mutant plants following germination and growth on solid 0.5×MS media (adjusted to pH 5.6) in the absence or presence of 50 mM NaCl. The mean±SE (*n* = 4–6) chlorophyll content is shown. ** (*P*<0.01) denotes significant difference between control and NaCl treatments as determined by one-way ANOVA.

The *cax1/cax2* and *cax2/cax3* double mutants showed no significant deleterious morphological phenotypes when germinated and grown on soil and looked equivalent to the *cax1*, *cax2* and *cax3* single mutants rather than the *cax1/cax3* mutant, which as shown previously [11], has a marked stunted phenotype on soil (Figure S1). However, on 0.5×MS agar medium, *cax2/cax3* had reduced growth (Figure S2), which was significantly different to wild type as determined by fresh weight (Figure 2A). The *cax1/cax2* and *cax2/cax3* mutants were also tested on a range of ion stress conditions: 25 mM CaCl_2_, 10 µM CdCl_2_, 10 mM LiCl, 50 mM NaCl, 25 mM MgCl_2_, and 1.5 mM MnCl_2_. The fresh weight of seedlings under these conditions was measured and a two-way ANOVA of all stress treatment data combined (Table S1) found that *cax1* and *cax1/cax2* were the only genotypes to consistently differ significantly from wild type (*P* = 0.007 and *P* = 0.001, respectively). When the metal treatments were evaluated individually, only two treatments identified significant differences among the genotypes as determined by one-way ANOVA: Mg and Mn stress. Under our conditions, we could not see any significant difference to fresh weight of any of the knockout genotypes compared to wild type in response to any of the other metal treatments including Ca stress or Na stress. Both *cax1* and *cax1/cax2* showed tolerance to 25 mM MgCl_2_ stress and grew several-fold better than wild type and any other genotype (Figure 2B). Growth phenotypes were also detected in mutants sown on 1.5 mM MnCl_2_ conditions. The *cax2* mutant showed increased sensitivity to Mn as demonstrated by reduced fresh weight compared to wild type, which was also seen in *cax2/cax3*, but when *CAX2* was deleted alongside *CAX1* (*cax1/cax2* mutant) the plants were not sensitive to Mn (Figure 2C), indicating that the loss of *CAX1* suppressed the *cax2* Mn sensitivity phenotype. Although treatment with 50 mM NaCl did not yield significant alterations in fresh weight, it did impact on total chlorophyll (Chl a+b) content in some of the mutants. Increased salt sensitivity in terms of reduced chlorophyll content compared to treatment without NaCl was found for *cax3* and *cax2/cax3* (Figure 3). The effects of the other metal treatments to leaf chlorophyll content were also measured but there were no significant differences found between genotypes and between stress and non-stress conditions.

### CAX gene mutation alters seed germination time

**Figure 4:**
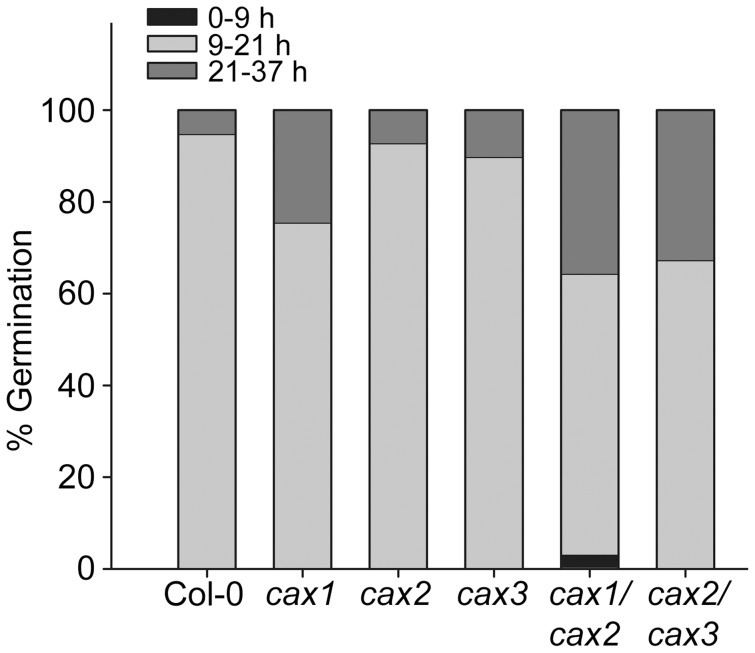
Germination profile of CAX mutant seedlings over three time periods. Approximately 100 seeds from Col-0 (wild type) and *cax* knockout mutant plants were sterilized and sown on 1% agar plates. After a 2-d incubation period at 4°C plates were moved to an environmentally-controlled growth chamber at 22°C. Radicle emergence from the testa was taken as the indicator of germination. Stacked bars indicate the percentage of seeds germinating 0–9 hours, 9–21 hours and 21–37 hours after transfer to 22°C. All values are corrected for seed non-viability, determined by quantifying the absence of seed germination after 10 d.

The CAX mutants were also examined for differences in germination. Approximately 100 seeds of each genotype were sown in a grid formation on 1% agar, stratified for 2 d in the dark at 4°C and then moved to an environmentally-controlled growth chamber. After a 9 h incubation period seeds were scored for radicle protrusion from the testa. The time-course of germination was divided into three phases for comparison: 0–9 h (‘early’), 9–21 h (‘intermediate’) and 21–37 h (‘late’), and the percentage of seeds germinating in each phase was calculated (Figure 4). Seeds from *cax1*, *cax1/cax2* and *cax2/cax3* germinated more slowly than wild type, with a greater percentage of seeds germinating in the late phase. The mean germination times of these three sets of mutant seeds ranged from 22.1 h to 25.3 h rather than 17.1 h to 19.2 h for the wild type, *cax2* and *cax3* seeds (Table 1). In order to further quantify the speed at which seeds germinate, the time required for 50% of viable seeds to germinate (T_50_) was calculated (Table 1). Wild type seeds germinated most quickly (T_50_ = 15.4 h), followed by single knockouts (T_50_ = ∼18 h), with the double knockout seeds the slowest (T_50_ = ∼21 h). Seeds were observed for a further 6 d and those found not to have germinated were considered non-viable. Maximum germination of the CAX mutant seeds ranged from 84.8% to 94.2%, with *cax1/cax2* seeds having the poorest viability compared to wild type with maximal germination of 96.7% (Table 1). Only the *cax2* knockout had a higher viability than wild type (97.1%).

**Table 1:** Germination time and maximum seed viability in CAX mutants.

Genotype	Time to 50% germination, h	Mean time to germinate, h	Maximum germination, %
Col-0	15.4±0.1	17.1±0.2	96.7
*cax1*	18.0±1.5	22.1±0.4	93.8
*cax2*	17.9±0.6	19.2±1.0	97.1
*cax3*	18.0±0.6	18.9±0.6	91.4
*cax1/cax2*	21.0±1.1	25.2±1.0	84.8
*cax2/cax3*	20.8±2.0	25.3±1.6	94.2

Seeds from Col-0 (wild type) and *cax* knockout mutant plants (approximately 70 of each line) were sterilized and sown on 1% agar before stratification for 2 d at 4°C in the dark. Plates were incubated (22°C, 16 h light/8 h dark) for 9 h then seeds were observed every 2 h for 12 h, then every 12 h until 10 d after which any seeds not germinated were considered non-viable. All values are the mean of three replicate experiments.

The wild type and mutant seeds were also examined under a range of excess metal conditions: 25 mM CaCl_2_, 10 µM CdCl_2_, 10 mM LiCl, 50 mM NaCl, 25 mM MgCl_2_ and 1.5 mM MnCl_2_. A two-way ANOVA identified a significant influence of the *cax1/cax2* genotype on seed viability, with these seeds being significantly less viable (*P* = 0.006) than wild type seeds and all other genotypes studied (Table S2). Establishment time, defined in this case as the time taken for the emergence of both cotyledons from the seed coat, was also measured. Mg, Mn and Li treatment had no significant effect on seedling establishment compared to no metal treatment, yet Ca treatment significantly delayed establishment in all seeds; however, no significant difference could be detected between any of the genotypes (Figure S3).

**Figure 5:**
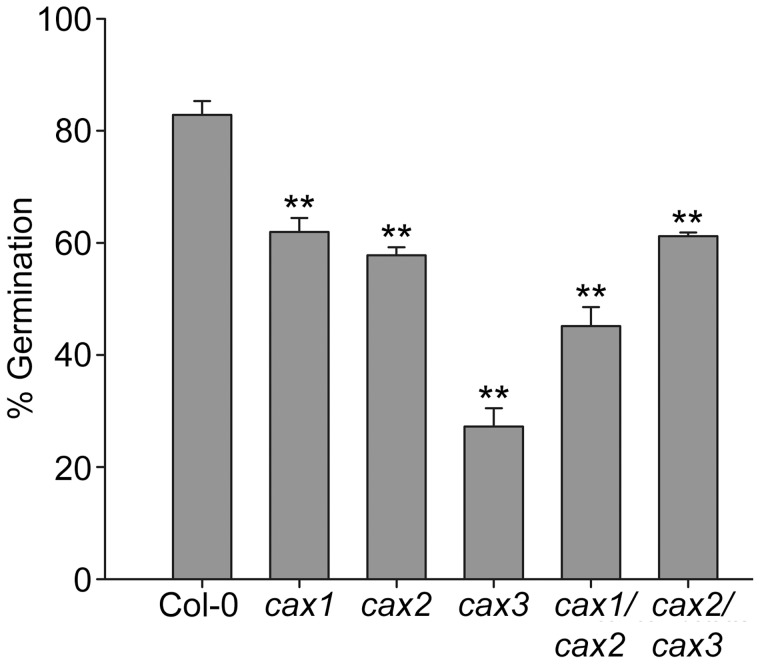
Germination of CAX mutant seedlings in response to abscisic acid (ABA). The germination of seeds from Col-0 (wild type) and *cax* knockout mutant plants was quantified after 24 h on 0.1 µM ABA. Approximately 100 seeds of each line were sterilized and sown on 1% agar plates containing ABA. After a 2-d incubation period at 4°C plates were moved to an environmentally-controlled growth chamber at 22°C. Radicle emergence from the testa was taken as the indicator of germination. Bars indicate the mean±SE percentage of germination. ** (*P*<0.01) denotes significant difference between CAX mutant lines and Col-0 control as determined by one-way ANOVA.

We previously found that *cax1* and *cax3* mutant seeds had reduced germination on media containing abscisic acid (ABA) when measured after 6 d [17]. In order to investigate the influence of ABA on germination of the double knockout mutants, seeds were sown on 1% agar containing ABA and scored for germination after 24 h. All of the single and double knockout mutants were found to have a significantly lower germination rate than wild type seeds on 0.1 µM ABA (Figure 5). Furthermore, at this early time point, the sensitivity to ABA by the *cax3* seeds particularly, and to a lesser extent the *cax1/cax2* seeds, could be differentiated from the other knockout seeds, including *cax1* and *cax2*.

### CAX mutant seeds have altered seed mineral nutrient contents

**Figure 6:**
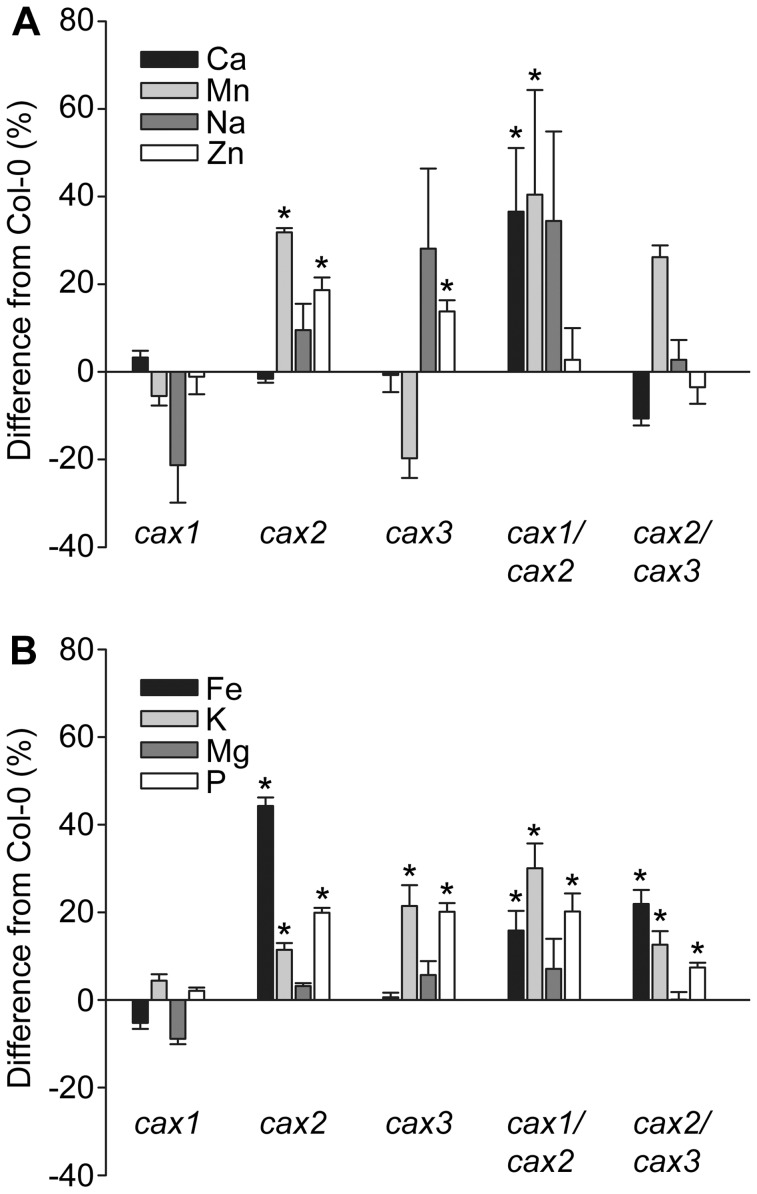
Difference in elemental concentration in dry seeds from CAX mutant plants. The concentrations of known or putative substrates of CAX transporters: Ca, Mn, Na and Zn (**A**) and of metals not thought to be transported by CAX: Fe, K, Mg and P (**B**) are shown as % difference from Col-0 (wild type) seeds. Dry seeds (approximately 15 mg per sample) were obtained from plants grown on soil without additional metal supplementation. Elemental analysis was performed by ICP-AES measurement. Bars indicate the mean±SE of three replicates. * (*P*<0.05) denotes significant difference from Col-0 as determined by one-way ANOVA.

Soil-grown wild type and mutant plants were subjected to ICP-AES element analysis to determine the mineral nutrient concentration in dried seeds (Figure S4) and leaves (Figure S5). Previously, we demonstrated minor nutrient concentration changes in the shoot tissue of *cax1* plants but more significant changes in *cax1/cax3* plants, particularly with respect to P, Mn and Zn [11]. Likewise, under the growth conditions of this study we observed significant leaf nutrient concentration changes in *cax1/cax3* compared to wild type: an increase in P and Zn, and a decrease in Na content (Figure S5). There was no change in leaf mineral concentration from the other CAX single and double knockout mutants compared to wild type. However, the seed mineral concentration profile was very different from that of the leaves (Figure S4). Due to the low number of *cax1/cax3* seed that was available from this poor seed-yielding plant, *cax1/cax3* seed elemental analysis was not performed, but seed from the other single and double mutants were examined. Ca^2+^, Mn^2+^, Na^+^ and Zn^2+^ have been demonstrated or indicated to be substrates of CAX1, CAX2 and CAX3 [15], [19], [25], [26]. Ca concentration was significantly increased by 37% in *cax1/cax2* seed compared to wild type (Figure 6A). *cax2* and *cax1/cax2* seed both had a significant increase in Mn concentration, by 32% and 40%, respectively, while Zn content was increased in *cax2* and *cax3* seed, by 19% and 14%, respectively. In all of the *cax2*, *cax1/cax2*, and *cax2/cax3* seed, Fe, K and P concentration was higher than in wild type, while K and P concentration was increased in *cax3* seed (Figure 6B).

### All CAX single and double knockouts show changes in cation/H^+^ exchange activity

In order to correlate the changes in CAX mutant metal stress response, seed germination and mineral nutrient concentration with vacuolar cation/H^+^ exchange activity, purified vacuolar-enriched membrane vesicles were isolated from wild type and knockout mutant seedlings. The isolated tonoplast-enriched vesicles were of high purity with low contamination from other membrane fractions, as determined by high inhibition of the nitrate- and bafilomycin-sensitive tonoplast V-ATPase and K^+^-dependent tonoplast H^+^-PPase activities (64–81% inhibition) and low inhibition of the vanadate-sensitive plasma membrane H^+^-ATPase and azide-sensitive mitochondrial H^+^-ATPase activities (11–12% inhibition) (Table S3). The purity and quality of membrane vesicles from each genotype were equivalent (Figure S6; data not shown).

**Figure 7:**
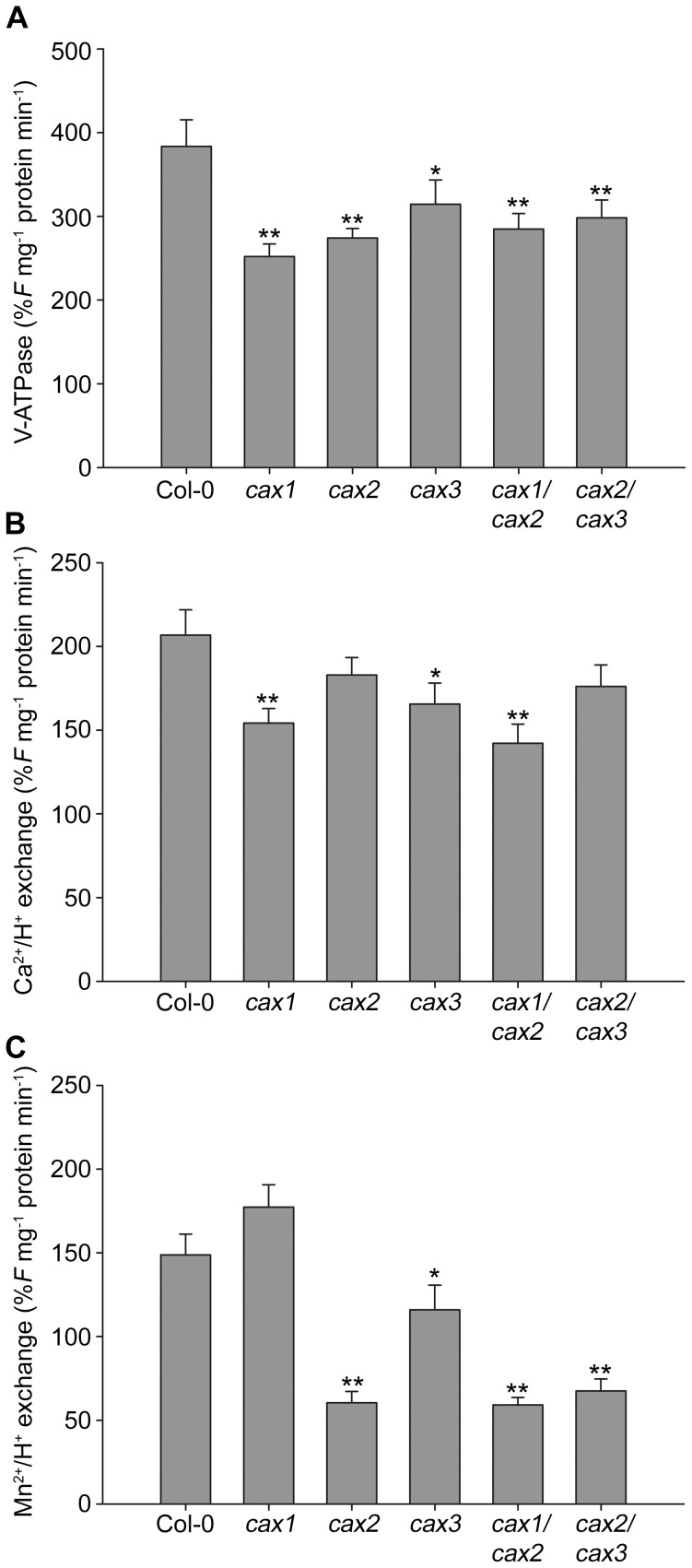
Vacuolar H^+^-ATPase, Ca^2+^/H^+^ and Mn^2+^/H^+^ exchanger activity in CAX mutant plants. (**A**) Initial rates of V-ATPase H^+^ transport activity in purified vacuolar-enriched membrane vesicles from Col-0 (wild type) and *cax* knockout mutant plants determined from the rates of acridine orange fluorescence quenching during the first 60 sec following the addition of Mg-ATP, as shown in Figure S6. (**B**) and (**C**) Initial rates of ΔpH-dependent cation/H^+^ exchange activity in purified vacuolar-enriched membrane vesicles from Col-0 and *cax* knockout mutant plants determined from the rates of acridine orange fluorescence recovery during the first 60 sec following the establishment of a steady-state pH gradient and the addition of 200 µM CaCl_2_ (**A**) or MnCl_2_ (**B**), as shown in Figure S6. *F* indicates relative fluorescence intensity. Membrane vesicles were prepared from 2-week-old plants grown on solid 0.5×MS media and pre-treated with 50 mM CaCl_2_ and 1.5 mM MnCl_2_ 14 h before harvest. The data represent means±SE from three experiments. ** (*P*<0.01) and * (*P*<0.05) denotes significant difference between CAX mutant lines and Col-0 control as determined by one-way ANOVA.

H^+^ pump activity into the vesicles and cation/H^+^ exchange activity (Ca^2+^- or Mn^2+^-dependent H^+^ flux out of the vesicles) was determined by the fluorescence quenching and recovery of acridine orange. The initial rates of fluorescence quenching and recovery can be used to quantify the activity of the V-ATPase and cation/H^+^ exchange [42]. The proton gradient across the tonoplast to drive the cation exchange is determined by the V-ATPase. Previously we have found that inhibition of CAX activity in *cax1*, *cax2* and *cax3* knockouts leads to a decrease in V-ATPase activity [11], [15], [25]. This may influence the rate of H^+^-dependent cation exchange, therefore V-ATPase activity for all lines was analysed. V-ATPase-dependent H^+^ pumping into the wild type and knockout membrane vesicles was initiated by the addition of Mg^2+^-ATP, and then fluorescence quenching was observed until a steady-state pH gradient was obtained, which could be completely abolished by protonophore addition (data not shown). Consistent with previous studies, V-ATPase proton pumping activity (Figure S6) was reduced in the single *cax1*, *cax2* and *cax3* mutants compared to wild type, as determined by the initial rates of H^+^ transport over 1 min (Figure 7A), and were also reduced in the *cax1/cax2* and *cax2/cax3* mutants. However, there was no significant difference in V-ATPase activity between single and double *cax* knockout lines. Determination of V-ATPase activity by measurement of ATPase hydrolytic activity found equivalent differences between wild type and the CAX mutant plants (data not shown).

Despite the difference in V-ATPase activity between wild type and *cax* knockout mutants, there was no significant difference in final steady state levels of pH gradient between any of the lines. Significant quench recovery, indicating dissipation of the pH gradient, following the addition of 200 µM CaCl_2_ or MnCl_2_ was measured in the tonoplast-enriched vesicles purified from wild type plants, indicative of Ca^2+^/H^+^ and Mn^2+^/H^+^ exchange activity (Figure S6). Ca^2+^/H^+^ exchange activity as determined by the initial rate of Ca^2+^-dependent fluorescence recovery over 1 min was significantly reduced in *cax1* and *cax3*, but not in *cax2* (Figure 7B). Mn^2+^/H^+^ exchange activity was markedly reduced in *cax2*, but not at all in *cax1* (Figure 7C). There was also a smaller reduction in Mn^2+^/H^+^ exchange activity in *cax3*. Ca^2+^/H^+^ exchange activity was significantly reduced in *cax1/cax2* but not in *cax2/cax3*, while a large reduction in Mn^2+^/H^+^ exchange activity was equivalent in both double knockout lines.

## Discussion

Previous studies have indicated a number of physiological roles for *CAX1, CAX2* and *CAX3* in mature *Arabidopsis* plants [5]. Although these genes are known to be expressed in seeds [11], [32], their function there remains unknown. Furthermore, previous work on the consequence of disrupting the function of *CAX* genes has been limited to single knockouts and one double knockout of two closely related genes (*cax1/cax3*) [11], [15], [25]. This work therefore represents the first in-depth study into the significance of CAX function on seed physiology, and the physiological consequences of simultaneously knocking out CAX genes from different sub-families.

The data shown here suggest a hitherto unidentified role for the CAX transport pathway in seed Ca^2+^ homeostasis, and thus an influence on seed physiology. In particular *cax1/cax2* seeds showed delayed germination time and a loss in seed viability (Figure 4; Table 1; Table S2), along with increased total Ca^2+^ concentration when compared to wild type and to all other mutants studied (Figure 6). This is especially interesting because analysis of CAX2 vacuolar transporter function suggests that the CAX2 phenotype does not directly influence the vacuolar Ca^2+^ transport of shoot tissue (Figure 7). It might be expected that a reduction in vacuolar Ca^2+^ sequestration through combined loss of CAX1 and CAX2 would lead to a reduction in Ca^2+^ concentration rather than the increase observed in the *cax1/cax2* seed. Although there was a net reduction in Ca^2+^/H^+^ transport activity in the *cax1/cax2* line in vegetative tissue, due to the large amount of tissue required it was not possible to measure transport activity in the *cax1/cax2* seed and thus determine whether Ca^2+^/H^+^ exchange activity was higher than in vegetative tissue such as through up-regulation of other CAX transporters. We have previously found that there is a degree of compensation for loss of CAX activity and that other vacuolar Ca^2+^ transporters including Ca^2+^-ATPases are up-regulated in *cax1* and *cax1/cax3* knockout backgrounds [12], [15]. We might therefore speculate that in the *cax1/cax2* seed there is greater up-regulation and over-compensation by other Ca^2+^ transporters compared to in other tissues, resulting in more Ca^2+^ accumulation. Similarly, a potential up-regulation of other metal transporters may explain the increased metal concentration in the *cax* knockout seed.

Although significant alterations in seed Ca concentration were not consistently observed in all mutants exhibiting delayed germination, it is important to note that analysis of total seed content alone does not allow for the influence of sub-tissue (and indeed sub-cellular) partitioning of Ca^2+^ in seeds. A recent study into the distribution of Ca and other mineral nutrients within seeds of *cax1*, *cax3* and *cax1/cax3* knockout mutants found that only subtle changes in total seed Ca could be detected, but more dramatic changes were observed in the Ca partioning between the seed coat and embryo [33]. Other previous studies have also indicated the importance of vacuolar mineral storage and transport in *Arabidopsis* seeds: in the developing seed, minerals are stored principally in the vacuoles of the embryo and the chalazal endosperm [43]. Furthermore, when vacuolar release of these minerals is impaired, such as in *nramp3* and *nramp4* vacuolar metal transporter knockout mutants, delays are seen in germination and early development [44]. Although there was no single metal change that correlated with a delay in germination within the data set in this study, it is possible that the increased total metal burden within the seeds had effects similar to external metal toxicity on the seeds and thus inhibited germination. However, we found that increased levels of external Ca in particular led to a delay in seedling establishment in all plants, although there was no difference between wild type and CAX mutant lines (Figure S3). Excess Ca also inhibits seedling development at the earlier germination stage and when Ca homeostasis is compromised such as in *cax1*, *cax3* and *cax1/cax3* backgrounds, germination in the presence of external Ca is further delayed [26]. Seed germination is a complex process depending on the combined functioning of many different processes; however, it has been suggested that a number of the reactions involved, such as ABA-mediated dormancy, are governed in part by cytosolic Ca^2+^ flux [45]–[47]. Germination was delayed in all CAX mutant lines (Table 1) and ABA-dependent germination inhibition was greater in all CAX lines compared to wild type (Figure 5). It is therefore tempting to speculate that perturbed CAX function in mutant seeds may lead to disruption in Ca^2+^ signalling dynamics through inhibited vacuolar Ca^2+^ uptake, which may in turn delay germination. Previous studies have shown that mutant *Arabidopsis* seeds lacking the vacuolar Ca^2+^ release channel TPC1 are insensitive to ABA-induced repression of germination and thus germinate more rapidly than wild type seeds [48].

Although originally identified as Ca^2+^ transporters, CAX1, CAX2 and CAX3 are now known to also be able to transport other ions including Mn^2+^ and Cd^2+^ [20], [23], [27]. Their specificities do not appear to be a consequence of the phylogenetic groupings, with CAX1 and CAX3 being members of the Type 1-A subfamily and CAX2 being a member of the Type 1-B subfamily [9]. The significance of these groupings is unknown, however, while knockouts of single CAX genes show very subtle phenotypes, plants lacking two CAX genes (*CAX1* and *CAX3*) were previously shown to exhibit a dramatic stunted phenotype, which is consistent with the equivalent and partially overlapping Ca^2+^ transport function of both transporters [11]. Although *CAX2* is up-regulated in the *cax1/cax3* plant, it is clearly unable to compensate [12], [15]. Here we show, in contrast, that knocking out CAX1 and CAX2 simultaneously does not result in obvious deleterious phenotypes on soil (Figure S1). There is however, a small but significant decrease in fresh weight in *cax2/cax3* mutants grown under controlled conditions on solid MS agar medium (Figure 2 and S2), suggesting slight perturbations to seedling growth.

Metals including Mg and Mn did not significantly affect seedling establishment (Figure S3) but did have an effect later in development of the plants. When grown in the presence of high external Mg, *cax1* and *cax1/cax2* seedlings were significantly more tolerant than wild type and all other mutants studied (Figure 2). Tolerance of *cax1* mutants to Mg has been previously reported [15], [28]. However, it is interesting to note that in this study *cax1/cax2* was phenotypically similar to *cax1* and not *cax2* (Figure 2B). This, more surprisingly, also appears to be the case when mutants were grown in the presence of high external Mn. Here *cax2* seedlings were significantly smaller than wild type seedlings, potentially due to vacuolar Mn^2+^ sequestration in the wild type and thus the Mn tolerance role of CAX2 [25], [49]. However, *cax1/cax2* seedlings are more phenotypically equivalent to *cax1* than *cax2* on Mn medium, and do not show any signs of Mn sensitivity, despite a reduction in Mn^2+^/H^+^ exchange activity (Figure 7). Indeed mutants lacking *CAX1* have previously been shown to display a degree of tolerance to excess Mn [15]. It appears therefore that the status of the *CAX1* gene dominates the physiology of the seedling in response to Mn stress. Furthermore, a statistical analysis of the impact of CAX mutation on the response to metal stress established that only *cax1* and *cax1/cax2* are consistently significantly different to wild type; indeed *cax1/cax2* was significantly different from all other genotypes studied except *cax1* (Table S1).

Other metal stresses did not affect the fresh weight of the single and double CAX mutant plants any differently than wild type. This included 25 mM Ca stress, which we previously found caused a slight increased reduction in growth of *cax3* seedlings compared to wild type [11]. However, under the experimental conditions of this study we did not observe any significant difference in fresh weight between wild type and mutants including *cax3* and *cax2/cax3*. Furthermore, no differences in plant fresh weight were found with the wild type and CAX mutant lines in response to NaCl stress but knockouts of *cax3* and *cax2/cax3* were found to contain significantly less chlorophyll when grown on media supplemented with NaCl (Figure 3). This corresponds with the genotypes with elevated levels of *CAX1* transcript (Figure 1), and we have also previously found that 35S-*CAX1* overexpression plants are more sensitive to NaCl [50]. Several studies implicate CAX proteins in Na^+^ transport [15], [26], [51], and CAX1 is thought to be activated by SOS2 (CIPK24), the protein kinase important for salt tolerance and ion homeostasis [50], [52]. Furthermore, NaCl stress can stimulate Ca^2+^ influx in the cytoplasm, while Na^+^ transport is in part regulated by a signalling cascade beginning with Ca^2+^ sensors such as CBL10 [53], [54]. Although it is not known to what extent alterations in vacuolar Ca^2+^ transporters are responsible for altered NaCl sensitivity it is possible that increased levels of CAX1, which lead to a reduction in cytosolic Ca^2+^ due to elevated vacuolar sequestration [55], perturb the normal cellular response to high NaCl.

In conclusion, the data presented here identify evidence of a complex system of cross-talk among members of the cation/H^+^ exchanger family and compensatory mechanisms accounting for the subtleties in phenotypes associated with loss of function of one family member with regard to the normal physiology of the plant. However, individual members of the family, notably CAX1, appear to have a more pronounced role in determining the physiology of the plant under conditions of metal stress. Additionally, evidence presented here indicates an important role for CAX proteins in seed germination and development, which may be linked particularly to maintenance of Ca homeostasis. Further work is required to elucidate the precise contribution of the family members to these processes and their potential applications.

## Supporting Information

Figure S1 **Morphological phenotype comparisons of the** ***cax1/cax2*** **and** ***cax2/cax3*** **double knockout mutant lines with the** ***cax1/cax3*** **line.** *cax* single and double knockout lines were grown on soil alongside Col-0 (wild type). Representative plants from each line are shown after 4 weeks growth under continuous light at 22°C. Only the *cax1/cax3* plants exhibit the stunted, leaf tip necrosis phenotype.
(TIF)

Figure S2 **Growth of CAX mutant plants on 0.5×MS agar plates.** Growth of Col-0 (wild type) and *cax* knockout mutant lines on solid 0.5×MS media (adjusted to pH 5.6). Representative plants from each line are shown after 21 days growth.
(TIF)

Figure S3 **Mean establishment time of CAX mutant seedlings in response to metal stress.** Seeds from Col-0 (wild type) and *cax* knockout plants (approximately 15 per line) were sterilized and sown on media with or without metal supplements (1.5 mM MnCl_2_, 10 mM LiCl, 25 mM MgCl_2_, or 25 mM CaCl_2_). Seeds were stratified for 2 d at 4°C and then incubated under a 22°C 16 h light/8 h dark cycle. Seeds were observed every 24 h under a dissection microscope and the mean time for the seedlings to establish (the time for both cotyledons to clearly emerge from the seed coat) was determined for each line, using the equation Σ(N*_t_*T)/Σn where N*_t_* is the number of seeds established at each time point, T is the time point, and n is the total number of seeds. Bars indicate the mean±SE of 4 replicate experiments. ** (*P*<0.01) denotes significant difference of Ca treatment from control (non-metal stressed) treatment, as determined by one-way ANOVA.
(TIF)

Figure S4 **Nutrient concentration of dry seeds from CAX mutant plants.** Dry seeds (approximately 15 mg per sample) were obtained from Col-0 (wild type) and *cax* knockout plants grown on soil without additional metal supplementation. Quantification of Ca (**A**), Fe (**B**), K (**C**), Mg (**D**), Mn (**E**), Na (**F**), P (**G**) and Zn (**H**) was performed by ICP-AES. Bars indicate the mean±SE of three replicates. ** (*P*<0.01) and * (*P*<0.05) denotes significant difference from Col-0 as determined by one-way ANOVA.
(TIF)

Figure S5 **Nutrient concentration of leaves from CAX mutant plants.** Leaves (approximately 15 mg per sample) were obtained from 4-week-old Col-0 (wild type) and *cax* knockout plants grown on soil without additional metal supplementation. Quantification of Ca (**A**), Fe (**B**), K (**C**), Mg (**D**), Mn (**E**), Na (**F**), P (**G**) and Zn (**H**) was performed by ICP-AES. Bars indicate the mean±SE of three replicates. ** (*P*<0.01) and * (*P*<0.05) denotes significant difference from Col-0 as determined by one-way ANOVA.
(TIF)

Figure S6 **V-ATPase-dependent quenching and cation-dependent recovery of acridine orange fluorescence in vacuolar-enriched membrane vesicles from CAX mutant plants.** Proton transport is shown in Col-0 (wild type) (**A**), *cax1* (**B**), *cax2* (**C**), *cax3* (**D**), *cax1/cax2* (**E**) and *cax2/cax3* (**F**) lines. Membrane vesicles were prepared from 2-week-old plants grown on solid 0.5×MS media and pre-treated with 50 mM CaCl_2_ and 1.5 mM MnCl_2_ 14 h before harvest. Proton pumping and generation of a pH gradient initiated by the addition of Mg-ATP at the time shown (arrow) was measured by the quenching of acridine orange fluorescence. When the steady-state pH gradient was obtained, 0.2 µM bafilomycin was added to inhibit V-ATPase activity. Immediately after this, Ca^2+^- and Mn^2+^-dependent dissipation of the pH gradient was measured by the recovery of acridine orange fluorescence following the addition of 200 µM CaCl_2_ (red line) or 200 µM MnCl_2_ (blue line) at the time shown (arrow). *F* indicates relative fluorescence intensity. Representative traces are shown from three experiments.
(TIF)

Table S1 Univariate statistical analysis of the fresh weight of CAX mutant plants in response to metal stress. The fresh weight of Col-0 (wild type) and *cax* knockout seedlings was determined 21 d after sowing on 0.5×MS (pH 5.6) medium supplemented with 25 mM CaCl_2_, 10 µM CdCl_2_, 10 mM LiCl, 50 mM NaCl, 25 mM MgCl_2_, 1.5 mM MnCl_2_, or no supplements, then incubation at 22°C under a 16 h light/8 h dark cycle. A two-way ANOVA with Tukey post-hoc test on the variance in fresh weight of mutant seedlings in response to metal stress identified the following significant differences (* *P*<0.05; ** *P*<0.01; *** *P*<0.001); *n* = 889.
(PDF)

Table S2 Univariate statistical analysis of the viability of CAX mutant seeds in response to metal stress. The viability of Col-0 (wild type) and *cax* knockout seeds was determined 10 d after sowing on 1% agar medium alone or supplemented with 25 mM CaCl_2_, 10 µM CdCl_2_, 10 mM LiCl, 50 mM NaCl, 25 mM MgCl_2_, or 1.5 mM MnCl_2_. Following sowing, seeds were stratified for 2 d at 4°C in the dark then incubated at 22°C under a 16 h light/8 h dark cycle. Any seeds not germinated after 10 d were considered non-viable. A two-way ANOVA with Tukey post-hoc test on the viability of seeds of the various mutants in response to metal stress identified the following significant differences (* *P*<0.05); *n* = 105.
(PDF)

Table S3 The effects of inhibitors on H^+^ pump hydrolytic activity in vacuolar-enriched membrane vesicles. Membrane vesicles were prepared from 2-week-old Col-0 (wild type) plants. ATPase activity (µmol Pi mg^−1^ protein h^−1^) at pH 8.0 (unless indicated) was measured in the presence of 2 mM ATP, 2 mM MgSO_4_ and 50 mM KCl. Pyrophosphatase (PPase) activity (µmol Pi mg^−1^ protein h^−1^) at pH 8.0 was measured in the presence of 0.1 mM pyrophosphate, 5 mM MgSO_4_ and 50 mM KCl. All reactions were performed over a 1 h incubation period at 37°C. Inhibitors of the plasma membrane H^+^-ATPase (vanadate), tonoplast H^+^-ATPase (nitrate and bafilomycin), mitochondrial ATPase (azide) and tonoplast K^+^-dependent H^+^-PPase (- KCl) were used. Mean activity (±SE) from three replicates is shown. n.d. – not determined.
(PDF)
