# Supplementary figures and images for: Knockout of Multiple Arabidopsis Cation/H+ Exchangers Suggests Isoform-Specific Roles in Metal Stress Response, Germination and Seed Mineral Nutrition
Source: PLoS One. 2012 Oct 12;7(10):e47455. doi: 10.1371/journal.pone.0047455 (PMC3470555; doi:10.1371/journal.pone.0047455)

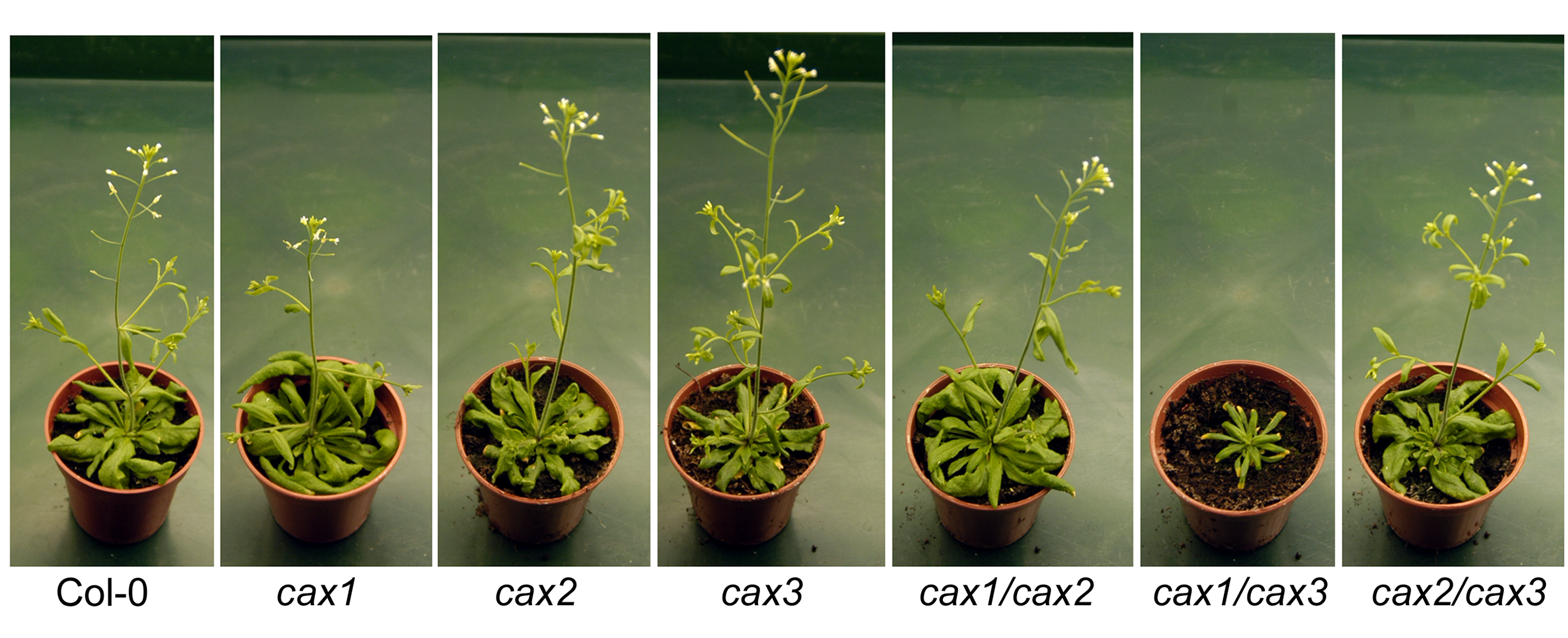

Supplement: Figure S1 — Morphological phenotype comparisons of the cax1/cax2 and cax2/cax3 double knockout mutant lines with the cax1/cax3 line. cax single and double knockout lines were grown on soil alongside Col-0 (wild type). Representative plants from each line are shown after 4 weeks growth under continuous light at 22°C. Only the cax1/cax3 plants exhibit the stunted, leaf tip necrosis phenotype. (TIF) [file pone.0047455.s001.tif]

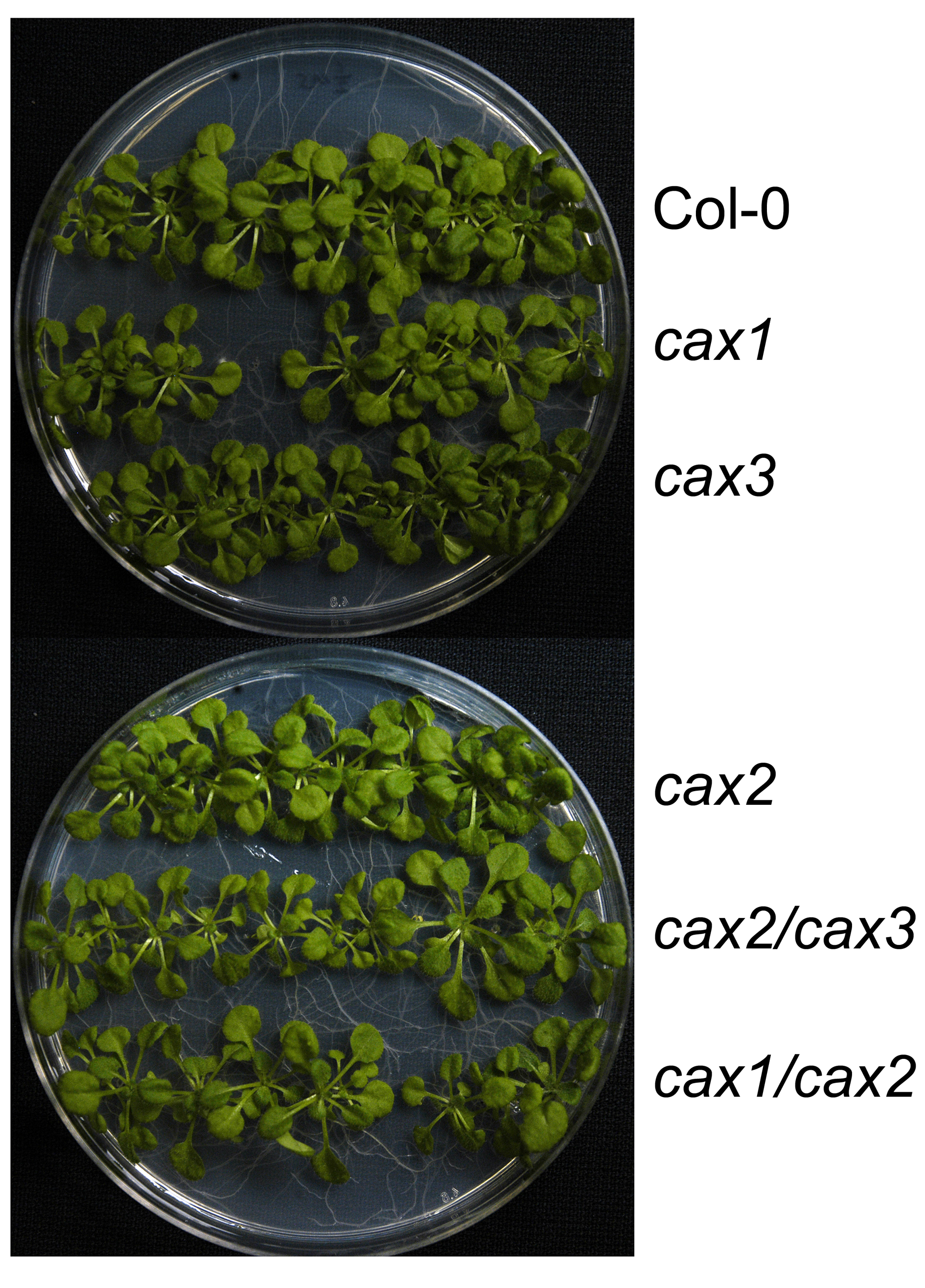

Supplement: Figure S2 — Growth of CAX mutant plants on 0.5×MS agar plates. Growth of Col-0 (wild type) and cax knockout mutant lines on solid 0.5×MS media (adjusted to pH 5.6). Representative plants from each line are shown after 21 days growth. (TIF) [file pone.0047455.s002.tif]

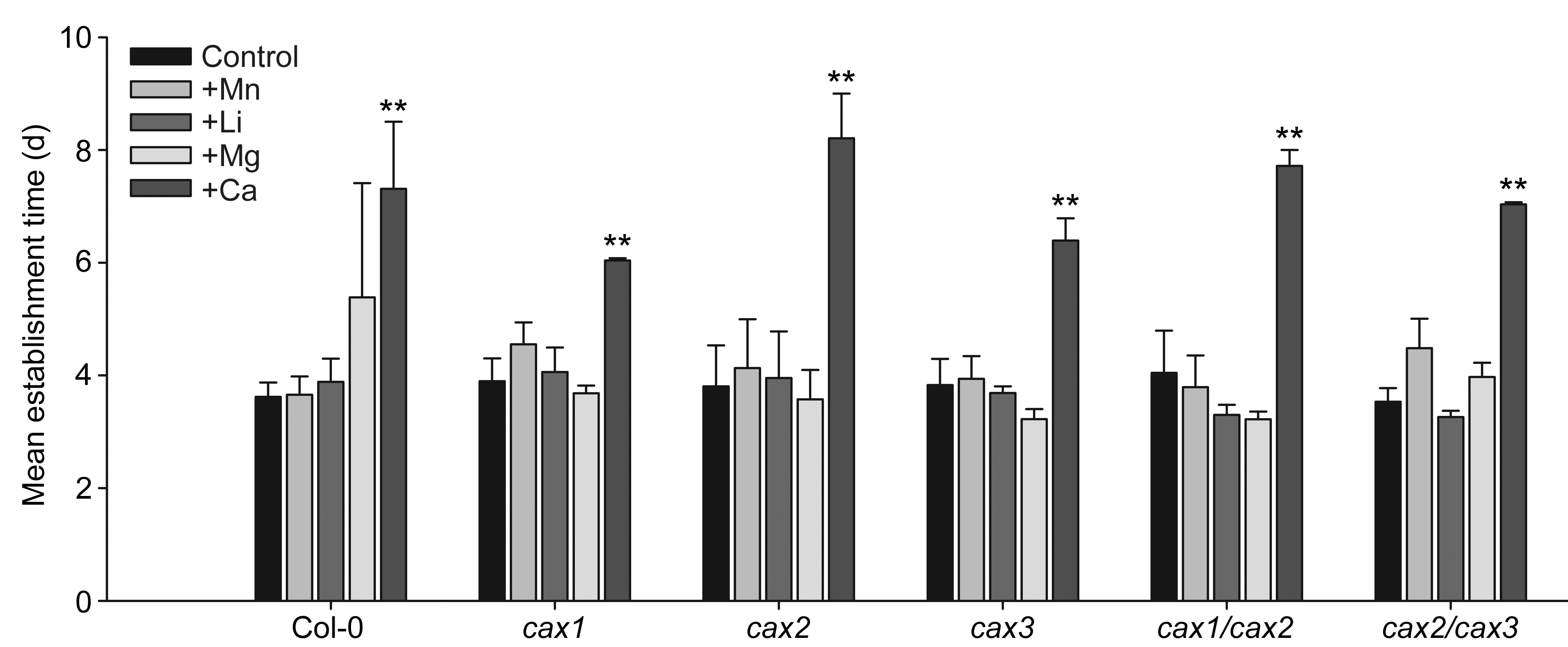

Supplement: Figure S3 — Mean establishment time of CAX mutant seedlings in response to metal stress. Seeds from Col-0 (wild type) and cax knockout plants (approximately 15 per line) were sterilized and sown on media with or without metal supplements (1.5 mM MnCl2, 10 mM LiCl, 25 mM MgCl2, or 25 mM CaCl2). Seeds were stratified for 2 d at 4°C and then incubated under a 22°C 16 h light/8 h dark cycle. Seeds were observed every 24 h under a dissection microscope and the mean time for the seedlings to establish (the time for both cotyledons to clearly emerge from the seed coat) was determined for each line, using the equation Σ(NtT)/Σn where Nt is the number of seeds established at each time point, T is the time point, and n is the total number of seeds. Bars indicate the mean±SE of 4 replicate experiments. ** (P<0.01) denotes significant difference of Ca treatment from control (non-metal stressed) treatment, as determined by one-way ANOVA. (TIF) [file pone.0047455.s003.tif]

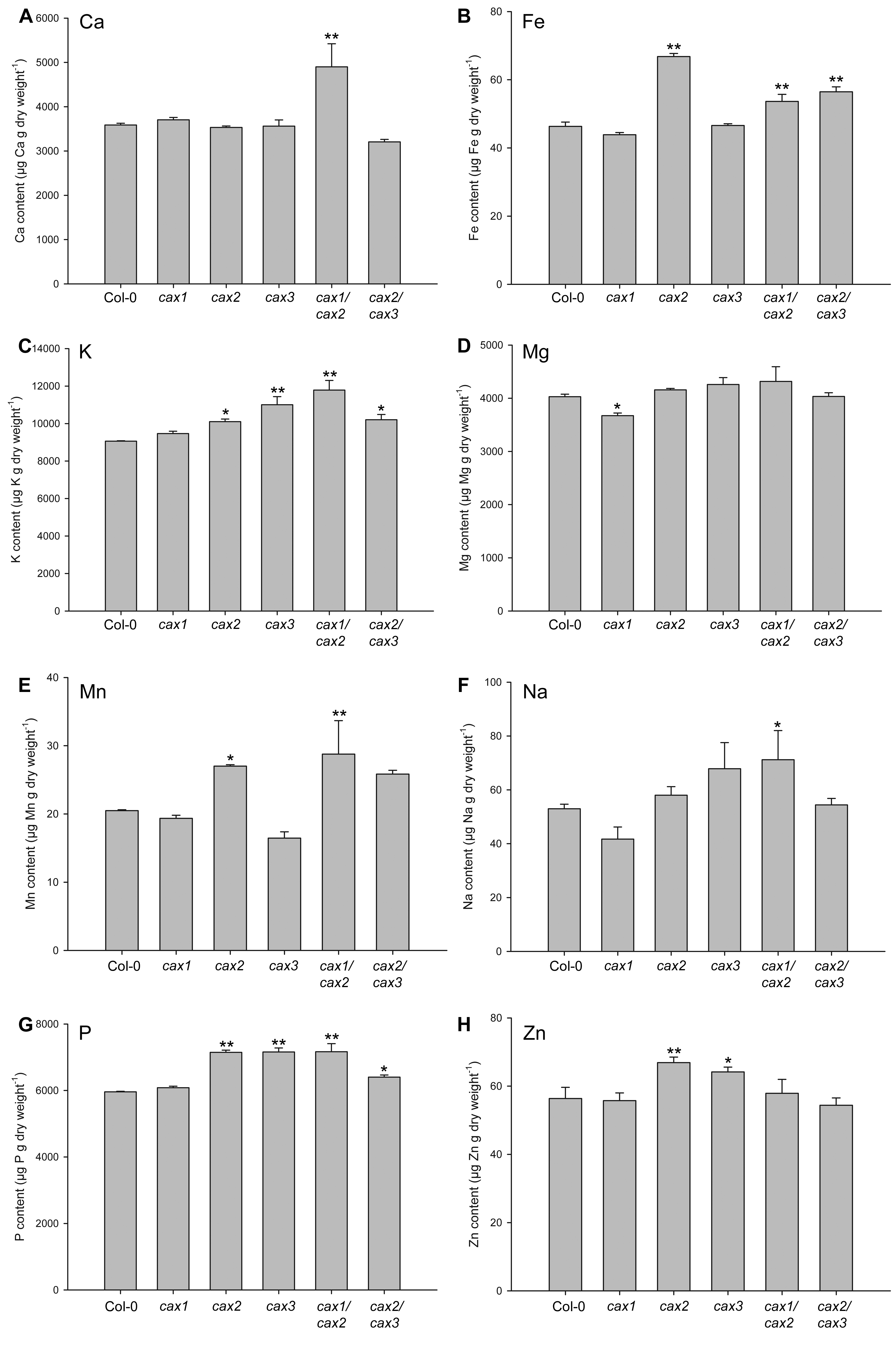

Supplement: Figure S4 — Nutrient concentration of dry seeds from CAX mutant plants. Dry seeds (approximately 15 mg per sample) were obtained from Col-0 (wild type) and cax knockout plants grown on soil without additional metal supplementation. Quantification of Ca (A), Fe (B), K (C), Mg (D), Mn (E), Na (F), P (G) and Zn (H) was performed by ICP-AES. Bars indicate the mean±SE of three replicates. ** (P<0.01) and * (P<0.05) denotes significant difference from Col-0 as determined by one-way ANOVA. (TIF) [file pone.0047455.s004.tif]

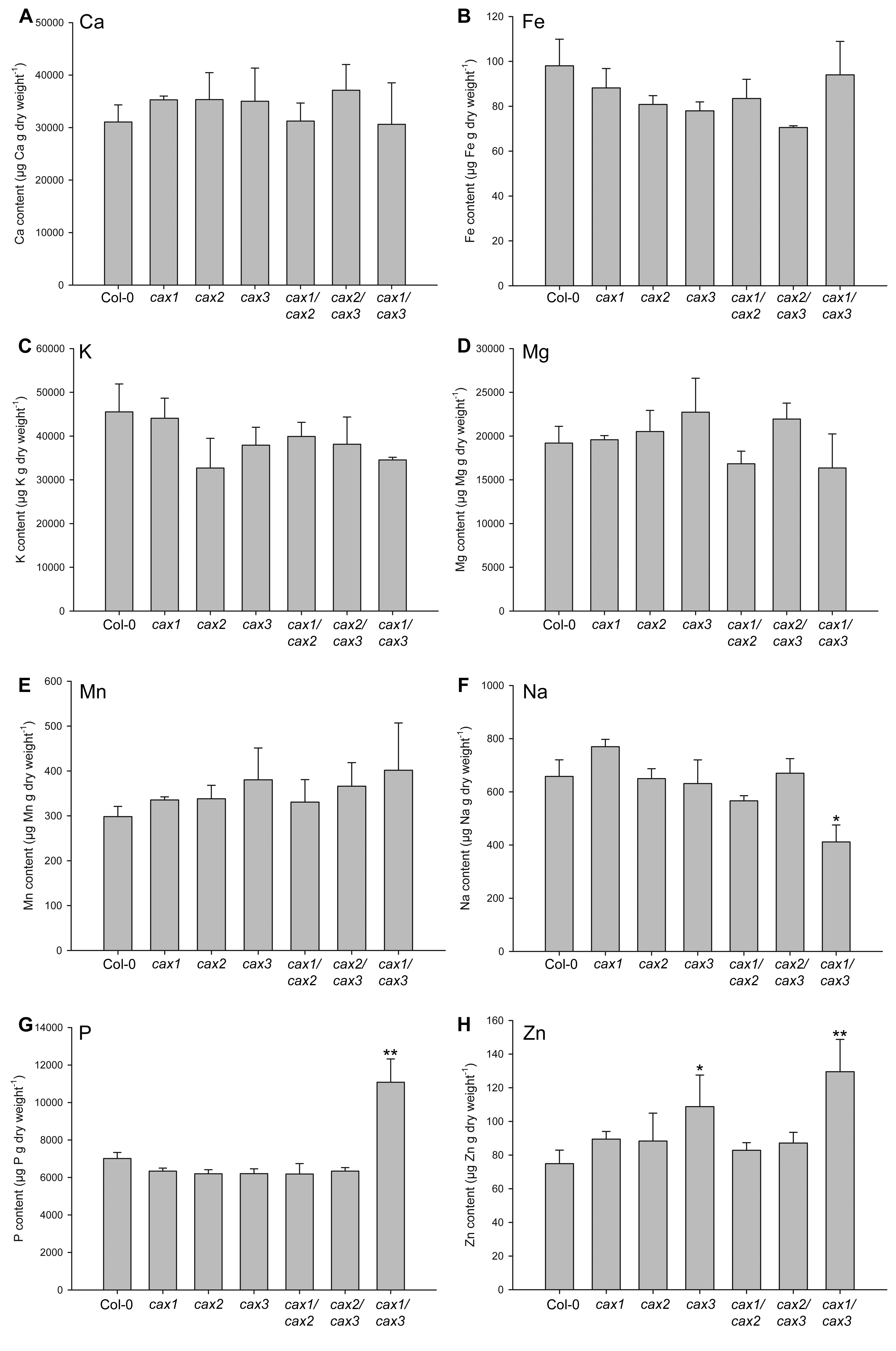

Supplement: Figure S5 — Nutrient concentration of leaves from CAX mutant plants. Leaves (approximately 15 mg per sample) were obtained from 4-week-old Col-0 (wild type) and cax knockout plants grown on soil without additional metal supplementation. Quantification of Ca (A), Fe (B), K (C), Mg (D), Mn (E), Na (F), P (G) and Zn (H) was performed by ICP-AES. Bars indicate the mean±SE of three replicates. ** (P<0.01) and * (P<0.05) denotes significant difference from Col-0 as determined by one-way ANOVA. (TIF) [file pone.0047455.s005.tif]

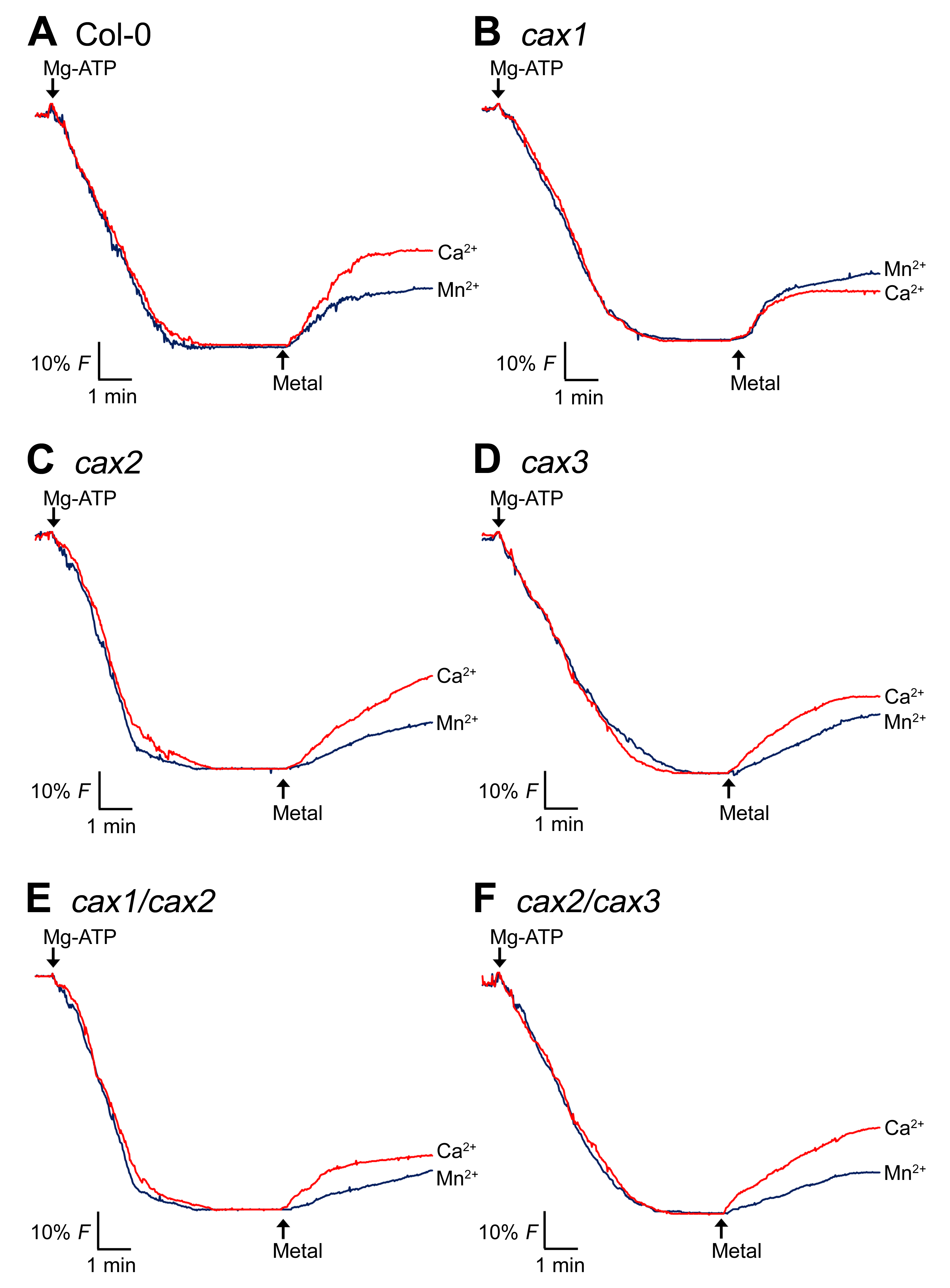

Supplement: Figure S6 — V-ATPase-dependent quenching and cation-dependent recovery of acridine orange fluorescence in vacuolar-enriched membrane vesicles from CAX mutant plants. Proton transport is shown in Col-0 (wild type) (A), cax1 (B), cax2 (C), cax3 (D), cax1/cax2 (E) and cax2/cax3 (F) lines. Membrane vesicles were prepared from 2-week-old plants grown on solid 0.5×MS media and pre-treated with 50 mM CaCl2 and 1.5 mM MnCl2 14 h before harvest. Proton pumping and generation of a pH gradient initiated by the addition of Mg-ATP at the time shown (arrow) was measured by the quenching of acridine orange fluorescence. When the steady-state pH gradient was obtained, 0.2 µM bafilomycin was added to inhibit V-ATPase activity. Immediately after this, Ca2+- and Mn2+-dependent dissipation of the pH gradient was measured by the recovery of acridine orange fluorescence following the addition of 200 µM CaCl2 (red line) or 200 µM MnCl2 (blue line) at the time shown (arrow). F indicates relative fluorescence intensity. Representative traces are shown from three experiments. (TIF) [file pone.0047455.s006.tif]
