# Supplementary material for: Knockout of Multiple Arabidopsis Cation/H+ Exchangers Suggests Isoform-Specific Roles in Metal Stress Response, Germination and Seed Mineral Nutrition
Source: PLoS One. 2012 Oct 12;7(10):e47455. doi: 10.1371/journal.pone.0047455 (PMC3470555; doi:10.1371/journal.pone.0047455)
Supplement: Table S1 — Univariate statistical analysis of the fresh weight of CAX mutant plants in response to metal stress. The fresh weight of Col-0 (wild type) and cax knockout seedlings was determined 21 d after sowing on 0.5×MS (pH 5.6) medium supplemented with 25 mM CaCl2, 10 µM CdCl2, 10 mM LiCl, 50 mM NaCl, 25 mM MgCl2, 1.5 mM MnCl2, or no supplements, then incubation at 22°C under a 16 h light/8 h dark cycle. A two-way ANOVA with Tukey post-hoc test on the variance in fresh weight of mutant seedlings in response to metal stress identified the following significant differences (* P<0.05; ** P<0.01; *** P<0.001); n = 889. (PDF) [file pone.0047455.s007.pdf]

Table S1. Univariate statistical analysis of the fresh weight of CAX mutant plants in response to metal stress. The fresh weight of Col-0 (wild type) and *cax* knockout seedlings was determined 21 d after sowing on 0.5× MS (pH 5.6) medium supplemented with 25 mM $CaCl_2$, 10 μM $CdCl_2$, 10 mM LiCl, 50 mM NaCl, 25 mM $MgCl_2$, 1.5 mM $MnCl_2$, or no supplements, then incubation at 22°C under a 16 h light/8 h dark cycle. A two-way ANOVA with Tukey post-hoc test on the variance in fresh weight of mutant seedlings in response to metal stress identified the following significant differences (* $P < 0.05$; ** $P < 0.01$; *** $P < 0.001$); $n = 889$.

**Genotype**	Col-0	*cax1*	*cax2*	*cax3*	*cax1/cax2*
Col-0					
cax1	**				
cax2					
cax3		***			
cax1/cax2	***		*	***	
cax2/cax3		***			***
