# Supplementary material for: Knockout of Multiple Arabidopsis Cation/H+ Exchangers Suggests Isoform-Specific Roles in Metal Stress Response, Germination and Seed Mineral Nutrition
Source: PLoS One. 2012 Oct 12;7(10):e47455. doi: 10.1371/journal.pone.0047455 (PMC3470555; doi:10.1371/journal.pone.0047455)
Supplement: Table S2 — Univariate statistical analysis of the viability of CAX mutant seeds in response to metal stress. The viability of Col-0 (wild type) and cax knockout seeds was determined 10 d after sowing on 1% agar medium alone or supplemented with 25 mM CaCl2, 10 µM CdCl2, 10 mM LiCl, 50 mM NaCl, 25 mM MgCl2, or 1.5 mM MnCl2. Following sowing, seeds were stratified for 2 d at 4°C in the dark then incubated at 22°C under a 16 h light/8 h dark cycle. Any seeds not germinated after 10 d were considered non-viable. A two-way ANOVA with Tukey post-hoc test on the viability of seeds of the various mutants in response to metal stress identified the following significant differences (* P<0.05); n = 105. (PDF) [file pone.0047455.s008.pdf]

Table S2. Univariate statistical analysis of the viability of CAX mutant seeds in response to metal stress. The viability of Col-0 (wild type) and *cax* knockout seeds was determined 10 d after sowing on 1% agar medium alone or supplemented with 25 mM $CaCl_2$, 10 μM $CdCl_2$, 10 mM LiCl, 50 mM NaCl, 25 mM $MgCl_2$, or 1.5 mM $MnCl_2$. Following sowing, seeds were stratified for 2 d at 4°C in the dark then incubated at 22°C under a 16 h light/8 h dark cycle. Any seeds not germinated after 10 d were considered non-viable. A two-way ANOVA with Tukey post-hoc test on the viability of seeds of the various mutants in response to metal stress identified the following significant differences (* $P < 0.05$); $n = 105$.

**Genotype**	Col-0	*cax1*	*cax2*	*cax3*	*cax1/cax2*
Col-0					
cax1					
cax2					
cax3					
cax1/cax2	*	*	*		
cax2/cax3					
