# Supplementary material for: Knockout of Multiple Arabidopsis Cation/H+ Exchangers Suggests Isoform-Specific Roles in Metal Stress Response, Germination and Seed Mineral Nutrition
Source: PLoS One. 2012 Oct 12;7(10):e47455. doi: 10.1371/journal.pone.0047455 (PMC3470555; doi:10.1371/journal.pone.0047455)
Supplement: Table S3 — The effects of inhibitors on H+ pump hydrolytic activity in vacuolar-enriched membrane vesicles. Membrane vesicles were prepared from 2-week-old Col-0 (wild type) plants. ATPase activity (µmol Pi mg−1 protein h−1) at pH 8.0 (unless indicated) was measured in the presence of 2 mM ATP, 2 mM MgSO4 and 50 mM KCl. Pyrophosphatase (PPase) activity (µmol Pi mg−1 protein h−1) at pH 8.0 was measured in the presence of 0.1 mM pyrophosphate, 5 mM MgSO4 and 50 mM KCl. All reactions were performed over a 1 h incubation period at 37°C. Inhibitors of the plasma membrane H+-ATPase (vanadate), tonoplast H+-ATPase (nitrate and bafilomycin), mitochondrial ATPase (azide) and tonoplast K+-dependent H+-PPase (- KCl) were used. Mean activity (±SE) from three replicates is shown. n.d. – not determined. (PDF) [file pone.0047455.s009.pdf]

Table S3. The effects of inhibitors on H^+ pump hydrolytic activity in vacuolar-enriched membrane vesicles. Membrane vesicles were prepared from 2-week-old Col-0 (wild type) plants. ATPase activity (µmol Pi mg^{-1} protein h^{-1}) at pH 8.0 (unless indicated) was measured in the presence of 2 mM ATP, 2 mM $MgSO_4$ and 50 mM KCl. Pyrophosphatase (PPase) activity (µmol Pi mg^{-1} protein h^{-1}) at pH 8.0 was measured in the presence of 0.1 mM pyrophosphate, 5 mM $MgSO_4$ and 50 mM KCl. All reactions were performed over a 1 hour incubation period at 37°C. Inhibitors of the plasma membrane H^+-ATPase (vanadate), tonoplast H^+-ATPase (nitrate and bafilomycin), mitochondrial ATPase (azide) and tonoplast K^+-dependent H^+-PPase (- KCl) were used. Mean activity (± SE) from three replicates is shown. n.d. – not determined.

**Treatment**	**ATPase activity**	**% inhibition**	**PPase activity**	**% inhibition**
Control	16.98 ± 1.23	0	19.20 ± 2.46	0
Vanadate (0.1 mM) at pH 8	15.82 ± 1.91	6.8	17.80 ± 1.20	7.3
Vanadate (0.1 mM) at pH 6.5	14.95 ± 1.72	12	n.d.	n.d.
Azide (1 mM)	15.06 ± 2.54	11.3	20.52 ± 2.16	-6.9
Nitrate (50 mM)	6.07 ± 0.11	64.3	17.43 ± 2.60	9.2
Bafilomycin (0.2 µM)	3.19 ± 0.07	81.2	18.87 ± 1.93	1.7
No KCl	9.43 ± 0.78	44.5	4.81 ± 0.21	74.9
